# Supplementary material for: A point-of-care diagnostic for drug-induced liver injury using surface-enhanced Raman scattering lateral flow immunoassay
Source: Nat Commun. 2025 Jul 6;16:6223. doi: 10.1038/s41467-025-61600-9 (PMC12228770; doi:10.1038/s41467-025-61600-9)
Supplement: Supplementary file 1 — Supplementary Information [file 41467_2025_61600_MOESM1_ESM.pdf]

Supplementary Information: A point-of-care diagnostic for drug-induced liver injury using surface-enhanced Raman scattering lateral flow immunoassay

SERS active conjugates

A schematic showing the synthesis of the gold-4,4-dipyridyl-silica-antibody-nanoparticles (Au-DIPY-SiO_2-Ab NP) conjugate is shown in Supplementary Figure 1.

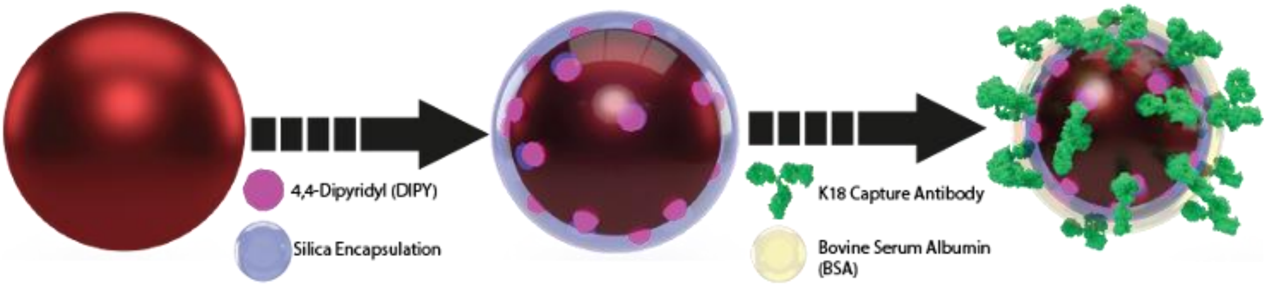

Supplementary Figure 1. Schematic showing synthesis of Au-DIPY-SiO_2-Ab NP conjugates. Au NP were functionalised with the Raman reporter DIPY and then encapsulated in a silica shell. The silica shell was then functionalised with K18 capture antibody and BSA. Au-DIPY-SiO_2-Ab NP, gold-4,4-dipyridyl-silica-antibody-nanoparticles; BSA, bovine serum albumin; K18, cytokeratin 18.

The Au NP must remain stable after the addition of SiO_2 and Ab to be used in the surface enhanced Raman scattering lateral flow immunoassay (SERS LFIA); therefore, every step of the synthesis was characterised using UV/Vis spectroscopy dynamic light scattering and using 785 nm laser excitation. UV/Vis spectroscopy characterisation provided information on the optical properties of the NP by producing an extinction spectrum, illustrated in Supplementary Fig. 2A. Analysis with a 785 nm laser measured the SERS signal produced by the NP, shown in Supplementary Fig. 2B. Data for the dynamic light scattering, a technique that measures the size of the NP, are presented in Supplementary Table 1. As expected, there was a change in the extinction spectra maxima, also known as the localised surface plasmon resonance (LSPR), between the Au NP and Au-DIPY-SiO_2 NP. This was due to the change in refractive index surrounding the NP secondary to the SiO_2 encapsulation. The size of the NP also increased after each addition, confirming the successful conjugation. A strong SERS signal was produced by the Au-DIPY-SiO_2 NP, which confirmed that the Raman reporter has bound to NP surface. The SERS signal was still present after the addition of Ab, with a small drop in SERS signal observed due to dilution. The characterisation data demonstrate that the

conjugates were stable, small enough in size to travel through the LFIA strip and produced an intense DIPY SERS signal, allowing them to be used and detected in the SERS-LFIA.

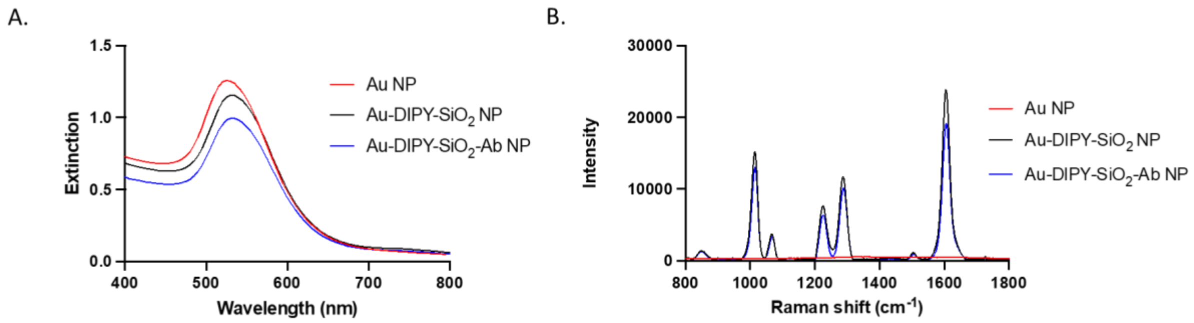

Supplementary Figure 2. UV/Vis spectroscopy and SERS characterisation of Au NP gives information about the optical and SERS properties of NP and assesses for any changes when SiO_2 and Ab were added. Extinction (A) and SERS spectra (B) of Au NP (red), Au-DIPY-SiO_2 NP (black) and Au-DIPY-SiO_2-Ab NP (blue). SERS spectra collected using 785 nm laser excitation, 80 mW laser power, 0.5 second acquisition. Ab, antibody; Au, gold; DIPY, 4,4-dipyridyl; NP, nanoparticle; SiO_2, silica; UV/Vis, ultraviolet-visible.

Supplementary Table 1 presents the results of the characterisation of the Au-DIPY-SiO_2-Ab NP used in the SERS LFIA and demonstrates that the conjugates were stable, small enough in size to travel through the LFIA strip and produced an intense DIPY SERS signal.

Supplementary Table 1. Characterisation data of Au-DIPY-SiO_2-Ab NP synthesis. LSPR-Localised surface plasmon resonance is a property of the extinction spectrum which gives information about the optical properties of NP and assesses for any changes when SiO_2 and Ab were added. Dynamic light scattering measures the size of the Au NP and provides information about NP size after the addition of SiO_2 and Ab. Analysing the NP with a 785 nm laser produces information on the SERS signal. It should remain strong after the addition of Ab. Ab, antibody; Au, gold; DIPY, 4,4 dipyridyl; LSPR, localised surface plasmon resonance; NP, nanoparticle; SERS, surface enhanced Raman scattering; SiO_2, silica.

**Sample**	**LSPR (nm)**	**Size (nm)**	**SERS Intensity at 1612 cm^{-1}**
Au NP	530	55	N/A
Au-DIPY-SiO_2 NP	535	65	25000
Au-DIPY-SiO_2-Ab NP	535	86	18000

Handheld Raman Reader

Both handheld Raman readers (HRR 3A and 4A) are comprised of a 785 nm laser excitation source, spectrometer module with CMOS detector, and sampling optics to direct and image laser light into a line on the LFA strip, which also directs and filters the Raman scattered light for detection by the spectrometer. It also integrates an LFIA cassette holder accessory to house the LFIA cassette during signal acquisition, with two functions:

1) Location and registering of the cassette for measurement of both control and sample lines, and
2) Enclosure of the cassette to mitigate possible exposure of the user to laser light.

The spectrometer module includes collimation and dispersion optics to spread incoming light by wavelength over a line array CMOS sensor. The diagram (Supplementary Figure 3) shows the optical path, isolated from the overall opto-electronics diagram. Light is emitted from the laser diode and passes through a Powell lens that images the Gaussian laser beam into a line of uniform intensity, which is then directed onto the sample by a dichroic long-pass mirror and focusing lens. The resulting Raman scattered light is collected by the same focusing lens, passes through the dichroic long-pass mirror and a long-pass dichroic filter to reject scattered laser light, and is directed through the slit focus assembly, a collimator assembly, and onto the diffraction grating. Light is diffracted through the grating, into the focusing lens assembly, and is directed onto the CMOS sensor for detection.

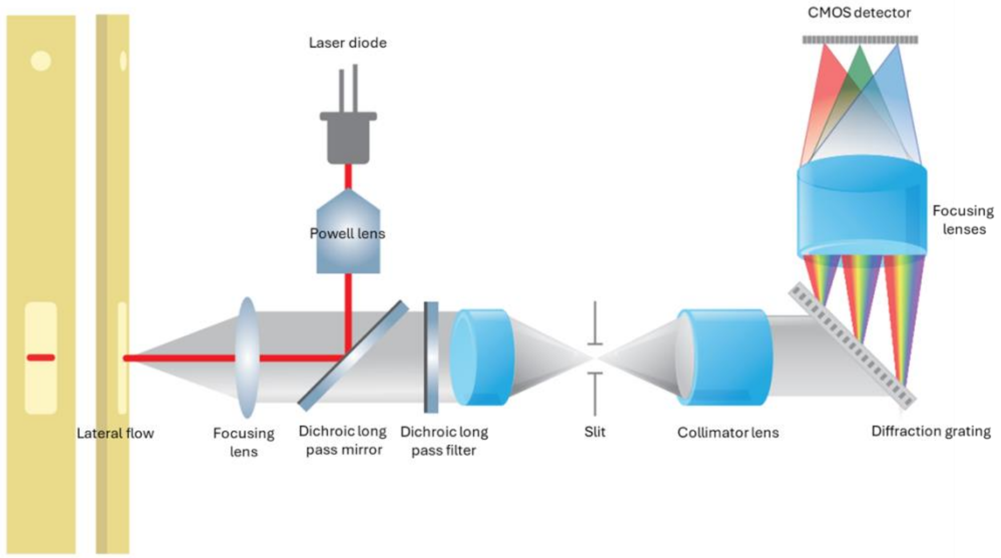

Supplementary Figure 3. Diagram of optical path through the handheld Raman reader (HRR) to the lateral flow immunoassay (LFIA) line. Technical illustrations were generated using SolidWorks CAD software.

Calibration curve developed for Study A

The calibration curve was produced in triplicate using from serum from three separate healthy donors to ensure robust results. Supplementary Fig. 4 represents the visual results from donor 1, obtained for the calibration curve developed for study A.

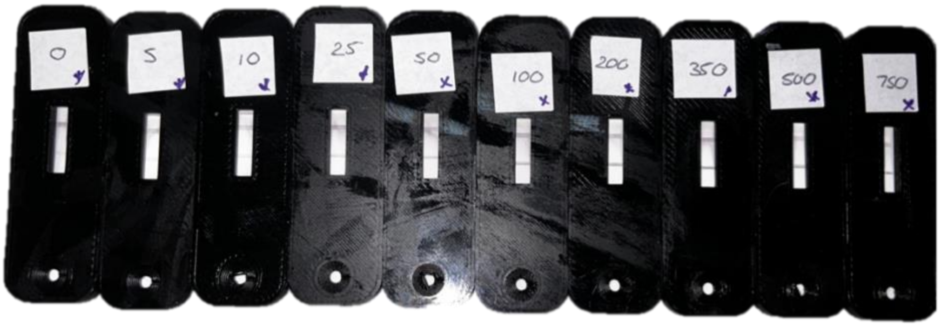

Supplementary Figure 4. Representative image of the calibration curve developed for study A. Serum samples were spiked with 0, 5 10, 25, 50, 100, 200, 350, 500, and 750 ng/mL of K18 (cytokeratin 18).

Test and control lines were analysed using a Wasatch Photonics (WP) HRR with a 3D printed accessory designed to hold the SERS-LFIA cassette in front of the laser (HRR version 3A). The average SERS signal from the test line obtained from the three healthy donors is presented in Supplementary Fig. 5A. As expected, an increase in the characteristic peaks of DIPY was observed as the concertation of K18 increased. This is due to the increase in the number of immunoassays and immobilised Au-DIPY-SiO_2-Ab NP. However, if the intensity of the 1612 cm^{-1} DIPY peak at each concentration is plotted, large variations are observed. This is illustrated in Supplementary Fig. 5B. The variation in SERS spectra at the test line is hypothesised to be due to a ‘serum effect’. Serum proteins can bind to the gold nanoparticle surface forming a protein corona with each serum sample producing its own unique corona. It is termed unique as volunteers have slightly different serum composition due to factors which influence protein production. This includes genetics, diet, health status and environmental impacts. The formation of the protein corona will impact the antibody-antigen interaction and in most cases, it reduces the non-specific binding[1]. It can also impact the flow of the nanoparticles through the LFIA strip as larger particles will travel slower through the strip. As different coronas are formed, the binding will be slightly different between volunteers. This is reflected in the SERS signal with higher binding corresponding to a higher SERS signal. Fortunately, this effect has been minimised by normalising the binding of the test line to the binding of the control line using the SERS spectra.

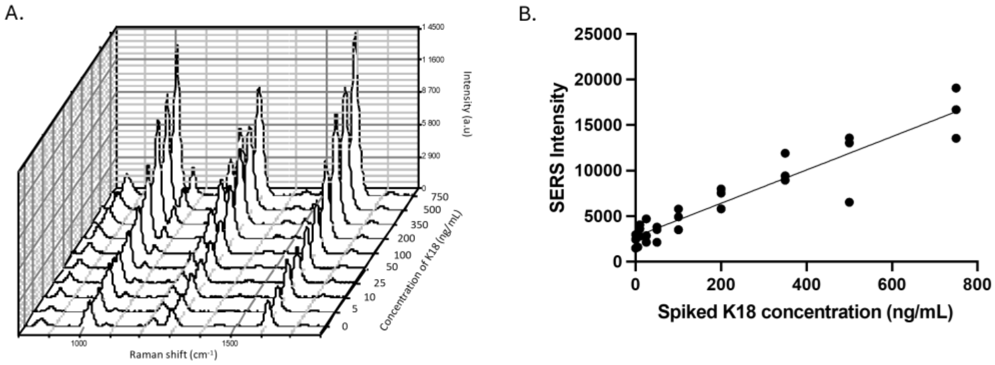

Supplementary Figure 5. A. Baselined SERS spectra obtained by averaging the signal of the test line from 3 healthy donors. B. Graph of spiked K18 concentration and the SERS intensity of the 1612 cm^{-1} peak of DIPY obtained from each donor's test line at that given concentration. The SERS signal was collected on the HRR 3A with a 785 nm laser excitation, 3.5 mW laser power, 1-second acquisition and 5 averages. Replicates of each test line were measured (n=3). Spectra baseline corrected on Wasatch Photonics (WP) software. A.U, arbitrary units; DIPY, 4,4 dipyridyl; *K18, cytokeratin 18; SERS, surface enhanced Raman scattering.*

As the test and control line binding are both affected by serum composition, standardising them against each other mitigates the 'serum effect'. We have chosen to standardise the output of the test and control line using a linear regression approach which produced a slope when the intensity of the test spectrum was plotted against the intensity of the control spectrum. As the concentration of K18 increased, so did the slope output. The calibration curve obtained using the linear regression approach is shown in Supplementary Fig. 6.

The linear regression method reduced the variation between donors and produced a linear calibration curve which was used to find the K18 concentration in patient samples in Study A.

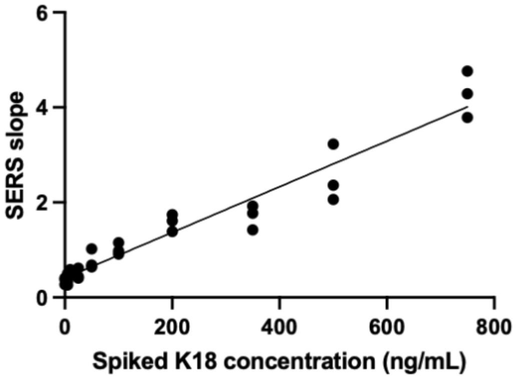

Supplementary Figure 6. Graph of spiked K18 concentration and the slope obtained when standardising the output from each donor at that given concentration (n=3 donors). K18, cytokeratin 18; SERS, surface enhanced Raman scattering.

Clinical Study A

To assess the sensitivity and specificity of the K18 SERS-LFIA, the novel assay was used to determine the concentration of K18 in 100 patient samples (50 with DILI and 50 without DILI). Each sample was run on the LFIA, analysed on the HRR, the slope output was calculated and the K18 concentration was determined by plotting the slope on the calibration curve (Supplementary Fig. 5B). Each sample was run in triplicate by three individual users and analysed on the HRR 3A three times. This resulted in 9 seperate K18 concentrations for each sample. The K18 concentrations were then uploaded to the Research Electronic Data Captire (REDCap) database (blinded to the DILI status) and analysed by Statisticians from Edinburgh Clinical Trials Unit.

The visual results of selected samples run as part of study A, along with their ALT, ELISA K18 and POC-DILI K18 values are shown in Supplementarty Fig. 7.

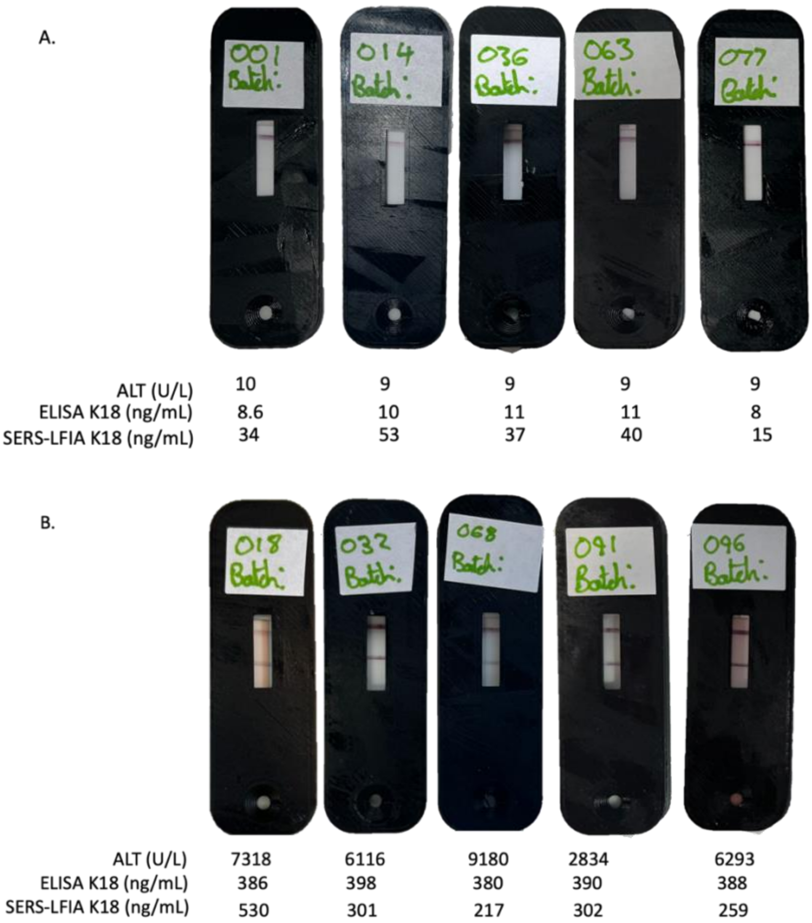

Supplementary Figure 7. Representative images of LFIAs from patients with the associated ALT values and K18 concentrations (calculated using ELISA and SERS LFIA). A) samples from participants without DILI and B) samples from participants with drug induced liver injury (DILI). ALT, alanine aminotransferase; ELISA, enzyme linked immunosorbent assay; K18, cytokeratin 18; LFIA, lateral flow immunoassay; SERS, surface enhanced Raman scattering.

Supplementary Table 2. K18 concentration using POC-DILI with the geometric mean of the K18 measurements obtained by analysers (n=3 measurements by 3 analysers for study A, n=3 measurements by one user for study B). DILI, drug-induced liver injury; GSD, geometric standard deviation.

	**Study A**						**Study B**	
	**Non-DILI**			**DILI**			**Non-DILI**	**DILI**
**Analyser**	W	X	Y	W	X	Y	Z	Z
**N**	50	50	50	49	49	49	50	50
**Geometric Mean (GSD)**	35 (1.94)	47 (1.89)	43 (1.78)	148 (1.74)	137 (1.91)	154 (1.84)	48 (2.51)	204 (1.53)
**Median [Q1, Q3]**	42 [21, 52]	53 [29, 68]	44 [31, 62]	149 [108, 225]	145 [98, 213]	157 [100, 240]	56 [34, 71]	213 [146, 200]
**Minimum, Maximum**	8, 180	11, 168	11, 145	29, 526	41, 625	44, 636	1, 208	81, 437

Study A and B - gender disaggregated data

To assess whether there was gender-based variation in the concentration of K18 in the clinical samples, using SERS-LFIA, the data was disagreggated. Supplementary Fig 8 presents female and male SERS-K18 concentrations for study A (primary and secondary statistical output) and study B.

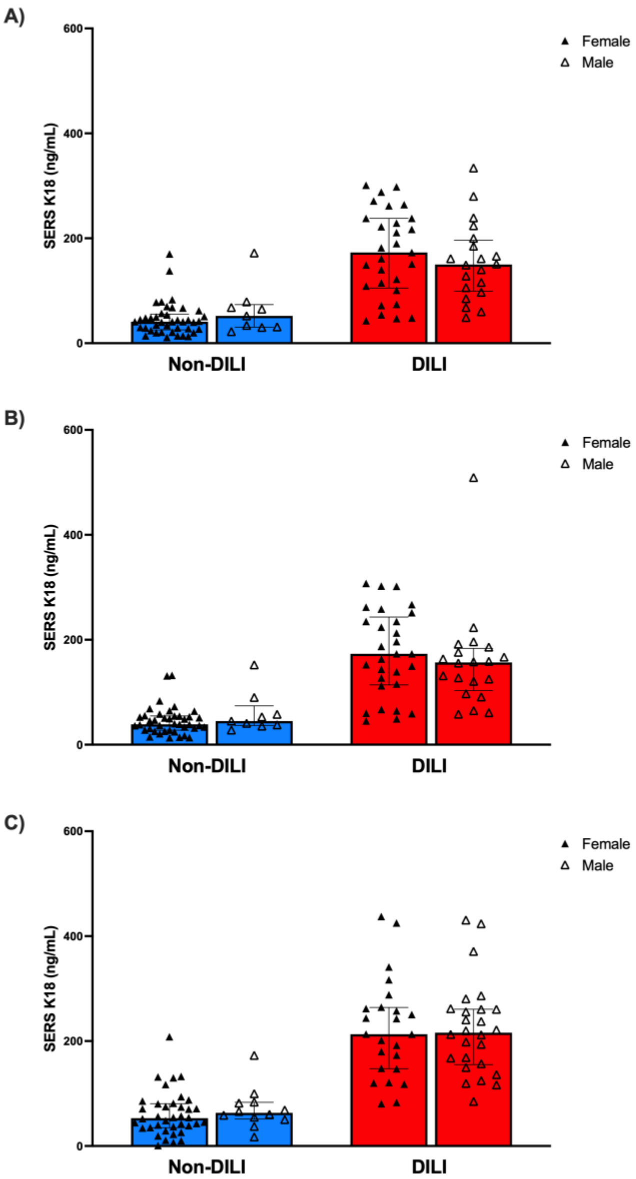

Supplementary Figure 8. Gender disaggregated data for clinical study A and B. A) Study A - Primary statistical output with randomly selected first values from the three analysers. B) Study A – geometric mean data points for the three analysers. C) Refined version of the diagnostic evaluated in Study B – mean data points for analyser. Bars represent the median values +/- interquartile range. Study A, n=99 and study B, n=100. DILI, drug-induced liver injury; K18, cytokeratin 18; SERS, surface enhanced Raman scattering.

Visual analysis

Three independent reviewers scored the DILI status of the test visually. The reviewers assigned a score of negative, if there was a weak test line when compared to the control, positive (+) if the test line was visible but weaker than the control or positive (++) if the test line was strong and had similar intensity as the control line (Supplementary Fig 9).

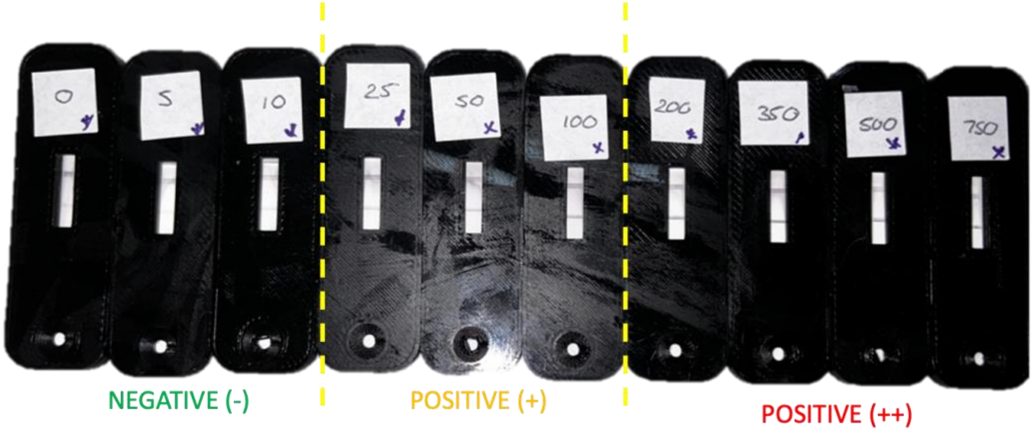

Supplementary Figure 9. Reference images for visual scoring of lateral flow immunoassays (LFIAs). The images are ranked from low K18 concentration to high concentration and categorised as negative (-), weakly positive (+) and positive (++).

Supplementary Table 3. Previous clinical studies demonstrating that K18 is a sensitive and accurate biomarker of DILI in large patient cohorts. The results of study A and B with the K18 SERS-LFIA are presented in red. DILI, drug-induced liver injury; K18, cytokeratin-18; LFIA, lateral flow immunoassay; SERS, surface enhanced Raman scattering.

**Study**	**Cause of liver injury**	**N**	**ROC-AUC (95% CI)**	**Reference**
Hepatology 2013[2]	Paracetamol	129	0.94 (0.87-1.00)	Hepatology 2013 PMID 23390034
MAPP[3]	Paracetamol	985	0.94 (0.89-0.99)	The Lancet Gastroenterology & Hepatology 2018 PMID 29146439
BIOPAR[3]	Paracetamol	202	0.93 (0.81-1.00)	The Lancet Gastroenterology & Hepatology 2018 PMID 29146439
Hepatology 2019[4]	Non-paracetamol DILI	689	0.95 (0.93-0.97)	Hepatology 2019 PMID 29357190
Edinburgh/Pfizer 2020[5]	Paracetamol	198	0.98 (0.95-0.99)	Toxicological Sciences 2021 PMID 33483742
Nature Communications[6]	Non-paracetamol DILI	142	0.96 (0.92-0.99)	Nature Communications 2023 PMID 36869085
Nature Communications[6]	Non-paracetamol DILI	75	0.97 (0.94-1.00)	Nature Communications 2023 PMID 36869085
Spanish DILI Registry[7]	Non-paracetamol DILI	109	0.96 (0.91-1.00)	British Journal of Clinical Pharmacology 2023 PMID 36965054
Study A	Paracetamol	99	0.95 (0.91-0.99)	
Study B	Paracetamol	100	0.97 (0.93-1.00)	

Supplementary Table 4. STARD (Standards for Reporting Diagnostic accuracy studies) checklist.

Section & topic	No	Item	Reported on page #
**Title or abstract**			
	**1**	Identification as a study of diagnostic accuracy using at least one measure of accuracy (such as sensitivity, specificity, predictive values, or AUC)	1 (Title and Abstract)
**Abstract**			
	**2**	Structured summary of study design, methods, results, and conclusions (for specific guidance, see STARD for Abstracts)	N/A
**Introduction**			
	**3**	Scientific and clinical background, including the intended use and clinical role of the index test	1 – 5 (Introduction)
	**4**	Study objectives and hypotheses	4 (Introduction)
**Methods**			
Study design	**5**	Whether data collection was planned before the index test and reference standard were performed (prospective study) or after (retrospective study)	8 (Results), 23 (Methods)
Participants	**6**	Eligibility criteria	23 (Methods)
	**7**	On what basis potentially eligible participants were identified (such as symptoms, results from previous tests, inclusion in registry)	23 (Methods)
	**8**	Where and when potentially eligible participants were identified (setting, location and dates)	23 (Methods)
	**9**	Whether participants formed a consecutive, random or convenience series	23 (Methods)
Test methods	**10a**	Index test, in sufficient detail to allow replication	24 – 25 (Methods)
	**10b**	Reference standard, in sufficient detail to allow replication	24 (Methods – ELISA)<br>24 (Methods – ALT)
	**11**	Rationale for choosing the reference standard (if alternatives exist)	24 (Methods)
	**12a**	Definition of and rationale for test positivity cut-offs or result categories of the index test, distinguishing pre-specified from exploratory	10 – 13 (Results) and 25 - 26 (Methods)
	**12b**	Definition of and rationale for test positivity cut-offs or result categories of the reference standard, distinguishing pre-specified from exploratory	25 (Methods)
	**13a**	Whether clinical information and reference standard results were available to the performers/readers of the index test	24 – 26 (Methods)
	**13b**	Whether clinical information and index test results were available to the assessors of the reference standard	24 – 26 (Methods)
Analysis	**14**	Methods for estimating or comparing measures of diagnostic accuracy	25 - 26 (Methods)
	**15**	How indeterminate index test or reference standard results were handled	25 - 26 (Methods)
	**16**	How missing data on the index test and reference standard were handled	25 - 26 (Methods)
	**17**	Any analyses of variability in diagnostic accuracy, distinguishing pre-specified from exploratory	25 (Methods)
	**18**	Intended sample size and how it was determined	24 (Methods)
**Results**			
Participants	**19**	Flow of participants, using a diagram	12 (Results – Fig. 4)
	**20**	Baseline demographic and clinical characteristics of participants	9 (Results – Table 1)
	**21a**	Distribution of severity of disease in those with the target condition	9 (Results – Table 1)
	**21b**	Distribution of alternative diagnoses in those without the target condition	N/A
	**22**	Time interval and any clinical interventions between index test and reference standard	N/A
Test results	**23**	Cross tabulation of the index test results (or their distribution) by the results of the reference standard	11 (Results – Fig. 3)
	**24**	Estimates of diagnostic accuracy and their precision (such as 95% confidence intervals)	13 (Results – Table 2)
	**25**	Any adverse events from performing the index test or the reference standard	N/A
**Discussion**			
	**26**	Study limitations, including sources of potential bias, statistical uncertainty, and generalisability	16 – 19 (Discussion)
	**27**	Implications for practice, including the intended use and clinical role of the index test	16 – 19 (Discussion)
**Other information**			
	**28**	Registration number and name of registry	23 (Methods – MAPP2 study)
	**29**	Where the full study protocol can be accessed	20 – 26 (Methods)
	**30**	Sources of funding and other support; role of funders	26 - 27 (Acknowledgements)

Supplementary References

1. de Puig, H., Bosch, I., Carré-Camps, M. & Hamad-Schifferli, K. Effect of the Protein Corona on Antibody-Antigen Binding in Nanoparticle Sandwich Immunoassays. *Bioconjug Chem* **28**, 230-238 (2017).
2. Antoine, D.J., *et al.* Mechanistic biomarkers provide early and sensitive detection of acetaminophen-induced acute liver injury at first presentation to hospital. *Hepatology* **58**, 777-787 (2013).
3. Dear, J.W., *et al.* Risk stratification after paracetamol overdose using mechanistic biomarkers: results from two prospective cohort studies. *Lancet Gastroenterol Hepatol* **3**, 104-113 (2018).
4. Church, R.J., *et al.* Candidate biomarkers for the diagnosis and prognosis of drug-induced liver injury: An international collaborative effort. *Hepatology* **69**, 760-773 (2019).
5. Llewellyn, H.P., *et al.* Evaluating the Sensitivity and Specificity of Promising Circulating Biomarkers to Diagnose Liver Injury in Humans. *Toxicological Sciences* **181**, 23-34 (2021).
6. Ravindra, K.C., *et al.* Tandem mass tag-based quantitative proteomic profiling identifies candidate serum biomarkers of drug-induced liver injury in humans. *Nat Commun* **14**, 1215 (2023).
7. Cueto-Sánchez, A., *et al.* Evaluation of diagnostic and prognostic candidate biomarkers in drug-induced liver injury vs. other forms of acute liver damage. *Br J Clin Pharmacol* **89**, 2497-2507 (2023).
