# Supplementary material for: A point-of-care diagnostic for drug-induced liver injury using surface-enhanced Raman scattering lateral flow immunoassay
Source: Nat Commun. 2025 Jul 6;16:6223. doi: 10.1038/s41467-025-61600-9 (PMC12228770; doi:10.1038/s41467-025-61600-9)
Supplement: Supplementary file 2 — Reporting Summary [file 41467_2025_61600_MOESM2_ESM.pdf]

nature portfolio

Corresponding author(s): Professor James Dear

Last updated by author(s): Jun 11, 2025

Reporting Summary

Nature Portfolio wishes to improve the reproducibility of the work that we publish. This form provides structure for consistency and transparency in reporting. For further information on Nature Portfolio policies, see our Editorial Policies and the Editorial Policy Checklist.

Statistics

For all statistical analyses, confirm that the following items are present in the figure legend, table legend, main text, or Methods section.

n/a	Confirmed	
☐	☒	The exact sample size (*n*) for each experimental group/condition, given as a discrete number and unit of measurement
☐	☒	A statement on whether measurements were taken from distinct samples or whether the same sample was measured repeatedly
☐	☒	The statistical test(s) used AND whether they are one- or two-sided<br>*Only common tests should be described solely by name; describe more complex techniques in the Methods section.*
☐	☒	A description of all covariates tested
☒	☐	A description of any assumptions or corrections, such as tests of normality and adjustment for multiple comparisons
☐	☒	A full description of the statistical parameters including central tendency (e.g. means) or other basic estimates (e.g. regression coefficient) AND variation (e.g. standard deviation) or associated estimates of uncertainty (e.g. confidence intervals)
☐	☒	For null hypothesis testing, the test statistic (e.g. *F*, *t*, *r*) with confidence intervals, effect sizes, degrees of freedom and *P* value noted<br>*Give P values as exact values whenever suitable.*
☒	☐	For Bayesian analysis, information on the choice of priors and Markov chain Monte Carlo settings
☒	☐	For hierarchical and complex designs, identification of the appropriate level for tests and full reporting of outcomes
☒	☐	Estimates of effect sizes (e.g. Cohen's *d*, Pearson's *r*), indicating how they were calculated

Our web collection on statistics for biologists contains articles on many of the points above.

Software and code

Policy information about availability of computer code

Data collection	Data was obtained using Enlighten 4.0.11 software and analysed using Microsoft Excel. For linear regression we used R Studio.
Data analysis	Statistical analysis for study A was performed and reported using SAS Software 9.4 and Stata 16.<br><br>Statistical analysis for study B was performed using Prism 10.0 (GraphPad).

For manuscripts utilizing custom algorithms or software that are central to the research but not yet described in published literature, software must be made available to editors and reviewers. We strongly encourage code deposition in a community repository (e.g. GitHub). See the Nature Portfolio guidelines for submitting code & software for further information.

Data

Policy information about availability of data

All manuscripts must include a data availability statement. This statement should provide the following information, where applicable:

- Accession codes, unique identifiers, or web links for publicly available datasets
- A description of any restrictions on data availability
- For clinical datasets or third party data, please ensure that the statement adheres to our policy

The data generated in this study are provided in the Supplementary Information/Source Data file. The raw SERS data used in this study are available on the Pure portal (DOI: 10.15129/8fe6d3bc-25e3-4d18-aab1-b5a42c7b6645).

Research involving human participants, their data, or biological material

Policy information about studies with human participants or human data. See also policy information about sex, gender (identity/presentation), and sexual orientation and race, ethnicity and racism.

Reporting on sex and gender	Gender is used in our study because our population (people who have deliberately self harmed) often includes a spectrim of gender identities. Table 1 contains our study demographics.
Reporting on race, ethnicity, or other socially relevant groupings	Ethnicity is reported in table 1. The majority of subject identify as White (Scottish) which represents the population of Edinburgh. We acknowledge the importance of generating data in more diverse populations in our paper discussion.
Population characteristics	The covariates are presented in table 1. Some covariates differ between cases and controls (such as overdose type). This is to be expected as patterns of overdose influence the risk of liver injury (case).
Recruitment	Samples were randomly selected from a biobank of patient samples (written informed consent provided). Figure 4 presents the STARD diagram which allows the reader to follow the flow of participants.
Ethics oversight	London - South East Research Ethics Committee (18/LO/0894).

Note that full information on the approval of the study protocol must also be provided in the manuscript.

Field-specific reporting

Please select the one below that is the best fit for your research. If you are not sure, read the appropriate sections before making your selection.

☒ Life sciences ☐ Behavioural & social sciences ☐ Ecological, evolutionary & environmental sciences

For a reference copy of the document with all sections, see nature.com/documents/nr-reporting-summary-flat.pdf

Life sciences study design

All studies must disclose on these points even when the disclosure is negative.

Sample size	Two blinded, clinical studies each of 100 patient samples were performed to assess the sensitivity and specificity of the new assay. The serum samples consisted of 50 with and 50 without DILI (sample size as per Clinical and Laboratory Standards Institute guidelines for assay development).
Data exclusions	1 patient sample was excluded before unblinding due to the sample being lipemic and not running correctly on the lateral flow test.
Replication	We include 2 cohorts of different patients to test reproducibility. Both studies were successful and the data are reported in the manuscript.
Randomization	No randomization. Samples were case-controlled for those with DILI and those without DILI.
Blinding	Team members analysing the assay (both visual and SERS analysis) were blind to the status of all samples (case or control). Their results were then uploaded to the Research Electronic Data Capture (REDCap) database (blinded to the DILI status) and analysed by Statisticians from Edinburgh Clinical Trials Unit.

Reporting for specific materials, systems and methods

We require information from authors about some types of materials, experimental systems and methods used in many studies. Here, indicate whether each material, system or method listed is relevant to your study. If you are not sure if a list item applies to your research, read the appropriate section before selecting a response.

Materials & experimental systems

n/a	Involved in the study	
☐	☒	Antibodies
☒	☐	Eukaryotic cell lines
☒	☐	Palaeontology and archaeology
☒	☐	Animals and other organisms
☐	☒	Clinical data
☒	☐	Dual use research of concern
☒	☐	Plants

Methods

n/a	Involved in the study	
☒	☐	ChIP-seq
☒	☐	Flow cytometry
☒	☐	MRI-based neuroimaging

Antibodies

Antibodies used	Recombinant anti-cytokeratin 18 antibody (capture and detection) and goat anti-rabbit IgG were purchased from Abcam (USA).<br><br>Abcam Anti-Cytokeratin 18 antibody [EPR20619-8] - BSA and Azide free (Capture) ab244688 Lot No. GR3450571-2 and GR3450571-3<br>Abcam Anti-Cytokeratin 18 antibody [EPR20619-219] - BSA and Azide free (Detector) ab244954 Lot No.1004604-1<br>Abcam Goat Anti-Rabbit IgG H&L (Control) ab6702 Lot no. GR3359542-16
Validation	All antibodies were purchased from commercial sources and were validated by the suppliers for the required species (human).<br><br>Antibody validation is part of the milestones pre-defined by our funder (MRC DPFS program).<br>Antibody performance (tested for every antibody batch):<br>• Via Western Blot: Signal from 100 ng/mL K18 in buffer relative to buffer-only negative control. ≥x2 (target)/ x1.5 (acceptable) greater than from buffer-only negative control.<br>• Via SERS: Signal from 100 ng/mL K18 in buffer relative to buffer-only negative control ≥x3 (target)/ x1.5 (acceptable) greater than from buffer-only negative control.

Clinical data

Policy information about clinical studies

All manuscripts should comply with the ICMJEguidelines for publication of clinical research and a completedCONSORT checklist must be included with all submissions.

Clinical trial registration	The biobank for samples ClinicalTrials.gov identifier: NCT03497104
Study protocol	Biobank samples - ClinicalTrials.gov<br><br>A pre-analysis Statistical Analysis Plan was written by the Edinburgh Clinical Trials Unit Senior Statisicians and is available for review if required.
Data collection	Analysis of samples collected and stored at -80 degrees (with temperature monitoring).
Outcomes	A pre analysis Statistical Analysis Plan was written by the Edinburgh Clinical Trials Unit Senior Statisicians and is available for review if required.

Plants

Seed stocks	N/A
Novel plant genotypes	*Describe the methods by which all novel plant genotypes were produced. This includes those generated by transgenic approaches, gene editing, chemical/radiation-based mutagenesis and hybridization. For transgenic lines, describe the transformation method, the number of independent lines analyzed and the generation upon which experiments were performed. For gene-edited lines, describe the editor used, the endogenous sequence targeted for editing, the targeting guide RNA sequence (if applicable) and how the editor was applied.*
Authentication	*Describe any authentication procedures for each seed stock used or novel genotype generated. Describe any experiments used to assess the effect of a mutation and, where applicable, how potential secondary effects (e.g. second site T-DNA insertions, mosiacism, off-target gene editing) were examined.*
